# Integrin/Fak/Src-mediated regulation of cell survival and anoikis in human intestinal epithelial crypt cells: selective engagement and roles of PI3-K isoform complexes

**DOI:** 10.1007/s10495-012-0713-6

**Published:** 2012-03-09

**Authors:** Marco Beauséjour, Dominique Noël, Sonya Thibodeau, Véronique Bouchard, Charlène Harnois, Jean-François Beaulieu, Marie-Josée Demers, Pierre H. Vachon

**Affiliations:** Département d’anatomie et de Biologie Cellulaire, Faculté de Médecine et des Sciences de la Santé, Université de Sherbrooke, Sherbrooke, QC J1H5N4 Canada

**Keywords:** Anoikis, Fak, HIEC, PI3-K, Src, Survival

## Abstract

In human intestinal epithelial crypt (HIEC) cells, the PI3-K/Akt-1 pathway is crucial for the promotion of cell survival and suppression of anoikis. Class I PI3-K consists of a complex formed by a catalytic (C) and regulatory (R) subunit. Three R (p85α, β, and p55γ) and four C (p110α, β, γ and δ) isoforms are known. Herein, we analyzed the expression of PI3-K isoforms in HIEC cells and determined their roles in cell survival, as well as in the β1 integrin/Fak/Src-mediated suppression of anoikis. We report that: (1) the predominant PI3-K complexes expressed by HIEC cells are p110α/p85β and p110α/p55γ; (2) the inhibition and/or siRNA-mediated expression silencing of p110α, but not that of p110β, γ or δ, results in Akt-1 down-activation and consequent apoptosis; (3) the expression silencing of p85β or p55γ, but not that of p85α, likewise induces Akt-1 down-activation and apoptosis; however, the impact of a loss of p55γ on both Akt-1 activation and cell survival is significantly greater than that from the loss of p85β; and (4) both the p110α/p85β and p110α/p55γ complexes are engaged by β1 integrin/Fak/Src signaling; however, the engagement of p110α/p85β is primarily Src-dependent, whereas that of p110α/p55γ is primarily Fak-dependent (but Src-independent). Hence, HIEC cells selectively express PI3-K isoform complexes, translating into distinct roles in Akt-1 activation and cell survival, as well as in a selective engagement by Fak and/or Src within the context of β1 integrin/Fak/Src-mediated suppression of anoikis.

## Introduction

Caspase-dependent apoptosis constitutes a complex and finely tuned process which performs crucial functions in development, tissue homeostasis and repair, as well as in the pathogenesis of several diseases [1–5]. It is now well understood that normal cells are intrinsically wired by default to undergo apoptosis and, consequently, require the input of signals in order to maintain apoptosis in a suppressed mode when not needed, or warranted [2–6]. Such critical cell survival signals are provided by various extracellular cues, including cell adhesion. To this effect, normal cells undergo caspase-dependent apoptosis by a process termed *anoikis* (a.k.a. “detachment-induced apoptosis”, “integrin-mediated cell death”) whenever a disruption, or loss, of integrin-mediated cell adhesion occurs [6–12]. In epithelial cells, those integrins belonging to the β1 subfamily are not only largely responsible for the establishment of a physical link between the extracellular matrix (ECM) and the cytoskeleton, but furthermore prevent the activation of the common anoikis pathway while at the same time driving the stimulation of various survival-promoting pathways [6–12]. Hence, depending on the cell and tissue context, signaling originating from β1 integrins to promote cell survival and anoikis suppression will often implicate focal adhesion kinase (Fak; p125^Fak^), Src (p60^Src^) and the phosphatidylinositol-3 kinase (PI3-K)/Akt-1 (PKB; p57^Akt-1^) pathway [6–8, 10–16].

The PI3-K/Akt signaling pathway is implicated in the regulation of various cell processes, including cell survival [16–20]. The class I PI3-K consists of a complex that is formed by a catalytic (C) and regulatory (R) subunit [16–20]. As a lipid kinase, PI3-K phosphorylates the substrate phosphatidylinositol (4,5) biphosphate (PIP_2_) to produce phosphatidylinositol (3,4,5) triphosphate (PIP_3_), which activates the effectors of the pathway (e.g. Akt) [16–20]. The engagement/activation of a PI3-K complex typically occurs through the recruitment/binding of an R subunit via its N-terminal SH2 domain, thus leading to a conformational change and consequent activation of its associated C subunit [16–20]. With regards to integrin signaling, both Fak and Src have been shown competent in recruiting/engaging PI3-K in such a manner, whether directly or indirectly, depending on the cell type studied [6, 8, 11–17]. Incidentally, three R (p85α, p85β, p55γ) and four C (p110α, p110β, p110γ, p110δ) isoforms are known for constituting class I PI3-K complexes [16–23]. To this effect, there is increasing evidence that: (i) PI3-K isoform complexes can be selectively expressed and/or activated according to the cell type; and (ii) these isoform complexes can furthermore perform selective roles in the regulation of cell processes not only depending on the tissue context, but as well within the same given cell type [16–23].

Much remains to be understood of the molecular determinants that regulate cell survival and death in intestinal epithelial cells (IECs), including their roles in the development of gastrointestinal disorders. In this respect, an improved comprehension of the specific signaling mechanisms that regulate survival and apoptosis/anoikis in normal human intestinal epithelial crypt (HIEC) cells is of direct relevance to the physiopathology of the gut, especially when considering that crypt alterations and epithelial apoptosis are regularly observed in inflammatory bowel disease, and that the persistence of aberrant crypt cells can lead to cancer [24–27]. In previous studies, we have shown that the PI3-K/Akt-1 pathway is not only critical for the survival of HIEC cells [28–32], but is furthermore engaged by integrin β1/Fak/Src-mediated signaling for anoikis suppression in a Fak- and Src-dependent manner [32, 33]. These observations are of interest, since a deregulation of Fak, Src and/or PI3-K/Akt survival signaling is often encountered in gastrointestinal cancers [14, 27, 34–39]—especially in the case of anoikis-resistant metastatic cells [14, 34–39]. Additionally, there is evidence that individual PI3-K R and/or C isoforms may be distinctively deregulated in gastrointestinal cancers [34, 38, 40, 41]. Hence, the still remaining open questions of the identity of the specific PI3-K isoform complexes that are responsible for driving HIEC cell survival, as well as those isoform complexes that are specifically engaged by integrin β1/Fak/Src-mediated signaling, have become quite germane.

Consequently, in the present study, we investigated the expression of PI3-K isoforms and their roles in the integrin β1/Fak/Src-mediated regulation of cell survival and anoikis suppression in HIEC cells. We report herein that HIEC cells selectively express PI3-K isoform complexes, translating into distinct roles in cell survival, as well as in a selective engagement by Fak and/or Src within the context of integrin β1/Fak/Src-mediated survival signaling.

## Materials and methods

### Materials

Specific antibodies directed against p125^Fak^, the phosphotyrosine397 activated form of p125^Fak^ (^pY397^p125^Fak^), the Src-phosphorylated tyrosine 576 and 577 residues of p125^Fak^ (^pY576/577^p125^Fak^), p57^Akt-1^, the phosphoserine473 activated form of p57^Akt-1^ (^pS473^p57^Akt-1^), p60^Src^, the phosphotyrosine418 activated form of p60^Src^ (^pY418^p60^Src^), and actin, were used as described previously [28–33, 39] and were purchased from Abcam (Cambridge, CA), Cell Signaling Technology (Beverly, MA) and/or Millipore (Etobicoke, ON, Canada). Also used were specific antibodies directed to the following PI3-K C or R isoform subunits: p110α (Millipore; Cell Signaling Technology), p110β (Millipore), p110γ (Cell Signaling Technology), p110δ (Millipore), p85α (Millipore), p85β (Abcam) and p55γ (Santa Cruz Biotechnology, Santa Cruz, CA). Note that the working functionality of each of the aforementioned PI3-K isoform antibody was verified and established by using protein lysates from granulocyte macrophage colony-stimulating factor (GMCSF)-stimulated human neutrophils (a kind gift from Patrick McDonald, Département de Médecine, Faculté de Médecine et des Sciences de la Santé, Université de Sherbrooke, Sherbrooke, QC, Canada), which express all PI3-K isoforms studied herein [42]. Other materials were purchased from Sigma (Oakville, ON, Canada) and/or Fischer Scientific (St-Laurent, QC, Canada), except where otherwise specified.

### Cell culture

The normal, non-transformed and non-immortalized HIEC-6 cells, which exhibit all the morphological and functional properties of in vivo proliferative/undifferentiated human crypt enterocytes, have been extensively characterized elsewhere (as examples, see [24, 28–32, 39, 43–48]). HIEC-6 cells were maintained and grown as already described [28–32, 39, 45, 47]. For experiments, cell cultures were maintained 24 h in medium without serum (controls) or with (i) 10 μM PIK-75 (Calbiochem, San Diego, CA), for the specific inhibition of p110α PI3-K activity; (ii) 10 μM TGX221 (Calbiochem), for the specific inhibition of p110β PI3-K activity; (iii) 10 μM AS605240 (Tocris Bioscience, Ellisville, MO), for the specific inhibition of p110γ PI3-K activity; (iv) 10 μM IC87114 (Calbiochem), for the specific inhibition of p110δ PI3-K activity; (v) 30 μM Ly294002 (Calbiochem), for the “pan” inhibition of PI3-K activity enacted by p110α-δ; (vi) 1 μM PF573228 (Tocris Bioscience), for the specific inhibition of Fak; (vii) 20 μM PP2 (Calbiochem), for the inhibition of Src; (viii) 100 μg/ml of the monoclonal antibody P4C10 (a kind gift of Erkki Ruoslahti, The Sanford-Burnham Medical Research Institute, LaJolla, CA), which inhibits the binding activity of the β1 integrin subunit [28–33]; or (ix) 100 μg/ml non-immune mouse IgGs (Sigma), as control for the P4C10 blocking antibody. The working concentrations of the inhibitors used were determined previously with dose–response assays (not shown). It is noteworthy that control cultures included exposure to the same solvent as that used for inhibitors and showed no significant differences with cultures maintained in serum-free medium only (not shown). For full anoikis, cells were kept in suspension 24 h (serum-free) in poly-2-hydroxyethyl methacrylate (polyHEMA)-coated dishes, as already described [28–30, 32, 33, 39].

### Caspase-activated DNAse (CAD)-mediated DNA laddering assays

DNA was isolated and the visualization of CAD-mediated internucleosomal DNA fragmentation (DNA laddering) on 2% agarose gels (20 μg DNA/lane) was performed as described elsewhere [28–33]. Note that the method used for DNA extraction employs Triton rather than SDS, thus leaving behind most intact genomic DNA [28–33, 49].

### In situ terminal deoxynucleotidyl transferase (TDT)-mediated dUTP nick-end labeling (ISEL) assays

Coverslip-grown HIEC cells were processed and ISEL was carried out as previously described [28–33, 39]. Evaluation of ISEL-positive cells counterstained with 4′,6-diamidino-2-phenylindole (DAPI) was performed as described elsewhere [28–33, 39]. Typically, apoptotic indices were compared to those of control cultures, ×100 (expressed as “% of control”).

### Fluorometric caspase-3 (CASP-3) activity assays

The CASP-3 fluorometric assay used herein is based on the hydrolysis of acetyl Asp-Glu-Val-Asp 7-amido-4-methylcoumarin (Ac-DEVD-AMC) by CASP-3, resulting in the release of the fluorescent 7-amino-4-methylcoumarin (AMC) catalysis product. The excitation and emission wavelengths of AMC are 380 nm and 430–460 nm, respectively. Cells from treated and non-treated/control cultures were solubilized in modified cold IP buffer (see below), from which phenylmethylsulfonyl fluoride was omitted. From each assayed sample, 30 μg proteins were added to 500 μl of freshly prepared CASP-3 reaction buffer (100 mM HEPES (pH 7.5), 20% glycerol and 5 mM dithiothreitol), followed by adding 2 μl of a 5 mM stock solution of Ac-DEVD-AMC (Calbiochem), and a subsequent 2 h incubation at 37°C. A blank, constituting of modified IP buffer, reaction buffer and Ac-DEVD-AMC, was likewise incubated in parallel to the reaction mixtures. After incubation, another 500 μl of reaction buffer was added and reactions (blank included) were read in a Hitachi S-2500 Spectrofluorometer (graciously made available to us by Martin Bisaillon, Département de Biochimie, Faculté de Médecine et des Sciences de la Santé, Université de Sherbrooke, Sherbrooke, QC, Canada), at an excitation wavelength of 380 nm. For each assay, the exact AMC emission apex within the 430–460 nm range was first determined with the blank, which in turn dictated at which emission wavelength the reactions were read. The relative CASP-3 activity was determined thereafter by applying the formula ∆F/F_0_ = (F_E_ − F_0_)/F_0_, where F_0_ = levels of emitted fluorescence by the blank, F_E_ = levels of emitted fluorescence by the assayed reaction, and ∆F/F_0_ = relative CASP-3 activity of the assayed reaction. In turn, the relative CASP-3 activity from treated cultures was compared to that of non-treated/controls, ×100 (expressed as “% of control”).

### Reverse transcriptase-polymerase chain reaction (RT-PCR)

Total RNA extraction and subsequent RT-PCR were carried out as described previously [29, 30]. Specific primers for the amplification of p110α, p110β, p110γ, p110δ, p85α, p85β, p55γ and actin were purchased from Invitrogen Life Techologies (Grand Island, NY). Controls for reactions were: (a) DNA without adding primers; and (b) primers without adding DNA (not shown) [29, 30]. Relative expression levels of PI3-K isoform mRNAs were determined by comparison with actin mRNA as a reference. Band intensities of amplified fragments were scanned and semi-quantified using an Alpha Imager 1200 Documentation and Analysis system (Alpha Innotech, San Leandro, CA), in order to establish the ratios “PI3-K isoform/Actin”.

### Western blotting (WB)

Cell cultures were lysed in sample buffer (2.3% SDS, 10% glycerol, and 0.001% bromphenol blue in 62.5 mM Tris–HCl (pH 6.8) containing 5% β-mercaptoethanol) and processed as described previously [28–33, 39]. Proteins were resolved by SDS-PAGE (50 μg proteins/lane), electrotransferred and probed as already described [28–33, 39]. Immunoreactive bands were semi-quantified with Scion Image (Scion, Frederick, MD), as already described [28–33, 39], and the relative expression levels of PI3-K isoforms were determined by comparison with actin as a reference, in order to establish the ratios “PI3-K isoform/Actin”.

### Immunoprecipitation (IP)/co-IP analyses and relative kinase activation assays

Cell cultures were lysed in cold IP buffer [50 mM Tris–HCl (pH 7.2), 150 mM NaCl, 1 mM dithiothreitol, 0.5 mM EDTA, 1% Nonidet P-40, 0.5% sodium deoxycholate, 0.1% SDS, 100 μM Na_3_VO_4_, 1 mM phenylmethylsulfonyl fluoride, 0.5 μg/ml leupeptin, 0.5 μg/ml aprotinin, 0.7 μg/ml pestadin, 40 mM β-glycerophosphate, and 10 mM Na_2_P_4_O_7_] and processed for IP as described previously [28–33, 39]. Immunoprecipitates were solubilized in sample buffer, resolved by SDS-PAGE and probed by WB (see above). Relative kinase activation analyses were performed as already described [28–33, 39]. Typically, immunoreactive bands were semi-quantified with Scion Image (Scion), as already described [28–33, 39], and the relative activated levels of kinases were established with the ratios phosphorylated kinase/total kinase, which in turn were compared to control cultures, ×100 (expressed as “% of control”).

### Small interference RNA (siRNA)-mediated expression silencing assays

siRNAs specifically directed against the mRNAs of p110α (sip110α), p110β (sip110β), p85α (sip85α), p85β (sip85β) or p55γ (sip55γ) were purchased from OriGene (Rockville, MD). A non-silencing control siRNA (siCNS) was purchased from Qiagen (Mississauga, ON, Canada). HIECs were transfected with either of each siRNA at a final concentration of 10 nM, according to the protocol described previously [45]. Three siRNAs for each isoform analyzed were tested. Only those siRNAs that resulted in a reduction of relative protein expression levels of at least 75% (as assessed by WB; see Fig. 4a as example) were used herein. 48 h following transfection, cells were processed for analyses. In some experiments, combinations of two siRNAs directed against PI3-K isoform subunits were used, still at a final concentration of 10 nM each. Under such circumstances, the control siCNS was used at a final concentration of 20 nM instead.

### Data processing

Results and values shown represent mean ± SEM for at least three (n ≥ 3) separate experiments and/or cultures. Statistically significant differences were determined by the Student t test, with SigmaSTAT (Systat Software, San Jose, CA). Data were compiled, analyzed and processed with Excel (Microsoft, Redmond, WA). Except otherwise specified, images from blots, gels and scans were processed with Vistascan (Umax Technologies, Fremont, CA), Photoshop (Adobe, San Jose, CA) and PowerPoint (Microsoft).

## Results

### HIEC cells selectively express PI3-K subunit isoforms and isoform complexes

**Fig. 1:**
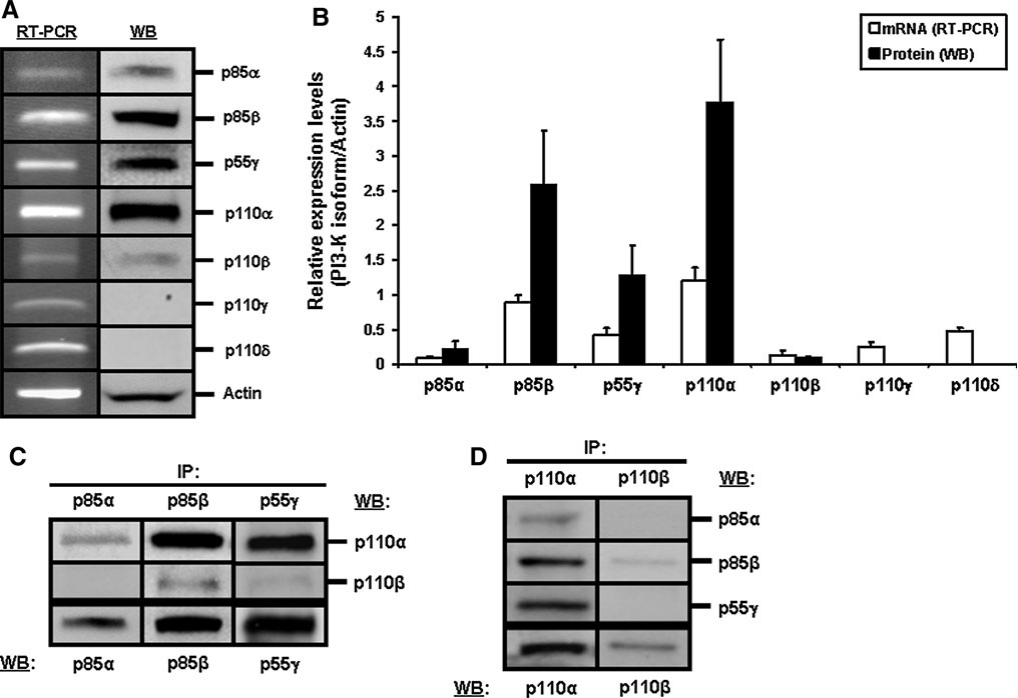
Expression of PI3-K isoforms and isoform complexes in HIEC cells. **a** Representative (n ≥ 3) RT-PCR (*left side of panel*) and WB (*right side of panel*) analyses of the expression of the known class I PI3-K C (p110α, p110β, p110γ, p110δ) and R (p85α, p85β, p55γ) isoforms, using isoform-specific primers (for RT-PCR) or antibodies (for WB). Actin expression was used as a reference. **b** Same as in (**a**), except that amplified bands (for mRNAs; *open columns*) and immunoreactive bands (for proteins; *filled columns*) were semi-quantified and compared to those of actin, in order to establish the relative expression levels for each isoform analyzed (n ≥ 3). **c** Representative (n ≥ 5) WB analyses of the IP of the PI3-K R isoforms p85α, p85β, and p55γ, for the verification of association by co-IP of the PI3-K C isoforms p110α and p110β, and consequent determination of the predominant PI3-K R/C isoform complexes expressed in HIEC cells. **d** Representative (n ≥ 3) WB analyses of the reciprocal validation of the IP/co-IP analyses in (**c**), this time via the IP of p110α and p110β, and verification of association of p85α, p85β, and/or p55γ

We first established the expression profile of class I PI3-K R and C isoforms in HIEC-6 cells. Semi-quantitative RT-PCR analyses indicate that these cells predominantly express the mRNAs for p85β, p55γ, p110α and p110δ, whereas those for p85α, p110β and p110γ are weakly expressed (Fig. 1a, left side of panel; 1b, open columns). Verification of protein expression by WB confirmed that p85β, p55γ and p110α are indeed predominantly expressed, and that p85α and p110β are effectively weakly expressed (Fig. 1a, right side of panel; 1b, filled columns). However, no protein products for either p110γ or p110δ were detected in HIEC-6 cells (Fig. 1a, right side of panel; 1b, filled columns), as confirmed by the strong detection of these two isoforms in GMCSF-stimulated human neutrophils using the same specific antibodies (not shown).

We then verified which PI3-K R/C isoform complexes are found in HIEC cells by performing IP analyses of R subunits and verification of association of C subunits via co-IP. As expected from our expression studies (see above), the IP of either p85β or p55γ revealed a strong association with p110α, but little to no association with p110β (Fig. 1c). Also as expected from our expression studies, what little of p85α that was IP yielded weakly detectable p110α and no detectable p110β (Fig. 1c). We confirmed these IP/co-IP observations by performing reciprocal analyses whereby C subunits were IP and the co-IP association of R subunits was verified (Fig. 1d).

Therefore, our expression profiling and IP/co-IP analyses altogether indicate that HIEC cells selectively express PI3-K R and C subunit isoforms, which in turn translates into a selective expression of PI3-K R/C isoform complexes. Specifically, p110α/p85β and p110α/p55γ are the largely predominant PI3-K isoform complexes found in these cells.

### Selective roles of PI3-K subunit isoforms and isoform complexes in HIEC cell survival

**Fig. 2:**
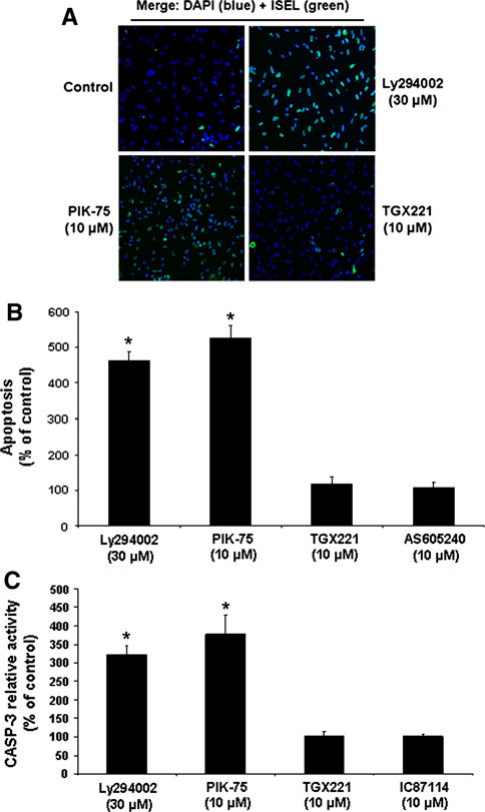
Selective roles of PI3-K C isoforms in the maintenance of HIEC cell survival. **a** Representative (n ≥ 4) double labeling-merged immunofluorescence micrographs of untreated HIEC cell cultures (control) and of cultures treated with Ly292002, PIK-75 or TGX221. ISEL (*green*) was thereafter performed, with DAPI (*blue*) counterstaining of nuclei. Original magnification: 20×. **b** HIEC cell cultures were maintained as in (**a**), in addition to being also treated with AS605240. ISEL was then performed. Statistically significant (0.0001 ≤ *P* ≤ 0.001) differences between treated and control cultures are indicated by (*). **c** HIEC cell cultures were maintained as in (**a**), in addition to being also treated with IC87114. CASP-3 relative activity was then established, using the substrate Ac-DEVD-AMC, by comparison to controls. Statistically significant (0.0005 ≤ *P* ≤ 0.005) differences between treated and control cultures are indicated by (*) (Color figure online)

**Fig. 3:**
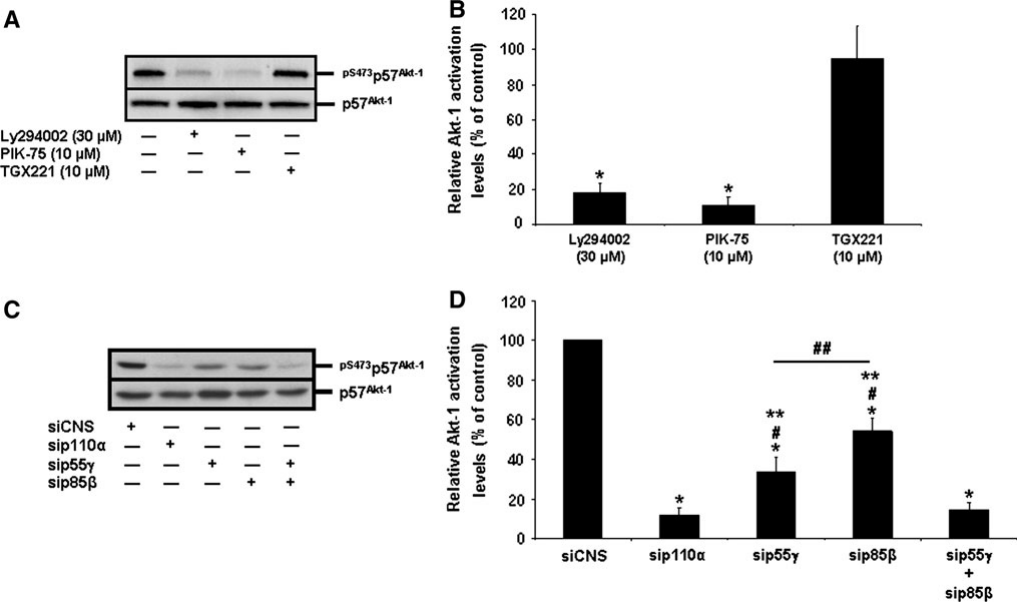
Distinct roles of PI3-K isoforms in the activation of Akt-1 in HIEC cells. **a** Representative (n ≥ 4) WB analyses of Akt-1 IP from untreated HIEC cell cultures (control) and from cultures treated with Ly292002, PIK-75 or TGX221. Specific antibodies for ^pS473^p57^Akt-1^ and p57^Akt-1^ were used. **b** Same as in (**a**), except that the relative activation levels of Akt-1 were established, then compared to controls. Statistically significant (0.0001 ≤ *P* ≤ 0.001) differences between treated and control cultures are indicated by (*). **c** Representative (n ≥ 3) WB analyses of Akt-1 IP from HIEC cells transfected with siCNS (control), sip110α, sip55γ, sip85β, or a combination of sip55γ + sip85β. Specific antibodies for ^pS473^p57^Akt-1^ and p57^Akt-1^ were used. **d** Same as in (**c**), except that the relative activation levels of Akt-1 were established, then compared to controls. Statistically significant (0.0001 ≤ *P* ≤ 0.001) differences between treated and control cultures are indicated by (*); statistically significant (0.0005 ≤ *P* ≤ 0.005) differences with sip110α are indicated by (#); statistically significant (0.0005 ≤ *P* ≤ 0.005) differences with the combination sip55γ + sip85β are indicated by (**); statistically significant (0.001 ≤ *P* ≤ 0.01) differences between sip55γ and sip85β are indicated by (##)

We functionally analyzed the roles of PI3-K isoforms in the maintenance of survival in HIEC-6 cells, considering that PI3-K and its main effector Akt-1 are critical for their survival [28–32]. Indeed, the “pan” inhibition of PI3-K activity results in extensive caspase-dependent apoptosis as assessed by ISEL (Fig. 2a, control vs. Ly294002; Fig. 2b, Ly294002) and CASP-3 activity (Fig. 2c, Ly294002), in addition to causing a sharp drop of Akt-1 activation (Fig. 3a, b, Ly294002). As expected from our PI3-K isoform expression profiling (see previous section), the specific inhibition of p110α similarly impacted upon HIEC cell survival (Fig. 2a–c, PIK-75) and Akt-1 activation (Fig. 3a, b, PIK-75), whereas the specific inhibition of p110β did not affect either (Figs. 2a–c, 3a–b, TGX221). Likewise, the specific inhibition of p110γ or p110δ failed to impact upon the survival of HIEC-6 cells (respectively Fig. 2b, AS60540; 2c, IC87114).

**Fig. 4:**
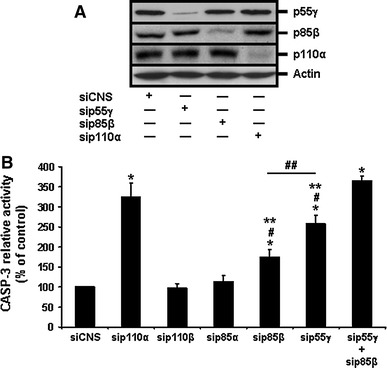
Selective roles of PI3-K C isoforms in the maintenance of HIEC cell survival. **a** Representative (n ≥ 4) siRNA-validating WB analyses of the expression of 55γ, p85β, and p110α, in HIEC cells transfected with siCNS (control), sip55γ, sip85β, or sip110α, using isoform-specific antibodies. **b** HIEC cells were transfected with siCNS (control), sip110α, sip110β, sip85α, sip85β, sip55γ, or a combination of sip55γ + sip85β. CASP-3 relative activity was then established, using the substrate Ac-DEVD-AMC, by comparison to controls. Statistically significant (0.0001 ≤ *P* ≤ 0.001) differences between treated and control cultures are indicated by (*); statistically significant (0.0005 ≤ *P* ≤ 0.005) differences between sip85β, or sip55γ, with sip110α are indicated by (#); statistically significant (0.0005 ≤ *P* ≤ 0.005) differences between sip85β, or sip55γ, with the combination sip55γ + sip85β are indicated by (**); statistically significant (0.001 ≤ *P* ≤ 0.01) differences between sip55γ and sip85β are indicated by (##)

We then performed siRNA-mediated specific PI3-K isoform expression silencing assays. Each siRNA used was verified for its isoform subunit specificity and its efficiency in reducing by at least 75% the expression of each targeted isoform. As shown in Fig. 4a as example, the siRNAs used against p110α (sip110α), p85β (sip85β) or p55γ (sip55γ) effectively reduced by more than 75% (semi-quantitative data not shown) the expression levels of their respective isoform subunit targets, as compared to the siCNS control, without affecting the expression of the other, non-targeted isoforms. As confirmation of our PI3-K activity inhibition experiments (see above), the sip110α severely impacted HIEC cell survival (Fig. 4b, sip110α vs. siCNS) and Akt-1 activation (Fig. 3c, d, sip110α vs. siCNS), whereas the sip110β failed to affect cell survival (Fig. 4b, sip110β vs. siCNS). Likewise, the sip85α did not significantly affect HIEC-6 cell survival (Fig. 4b, sip85α vs. siCNS). By stark contrast, the sip85β and sip55γ each significantly caused apoptosis (Fig. 4b, sip85β vs. siCNS, sip55γ vs. siCNS) and a drop in Akt-1 activation (Fig. 3c, d, sip85β vs. siCNS, sip55γ vs. siCNS). Interestingly, the impacts on CASP-3 activity and Akt-1 activation that resulted from either sip85β or sip55γ were significantly less than those produced by sip110α (Figs. 3c–d, 4b, sip110α vs. sip85β, sip110α vs. sip55γ). Additionally, the impacts on cell survival and Akt-1 activation by the sip85β were significantly less than those enacted by the sip55γ (Figs. 3c, d, 4b, sip85β vs. sip55γ). However, only when sip85β and sip55γ were used in combination did the resulting effects on CASP-3 activity and Akt-1 activation were significantly greater than those produced by either siRNA singly (Figs. 3c, d, 4b, sip85β + sip55γ vs. sip85β, sip85β + sip55γ vs. sip55γ) and, furthermore, did not differ significantly from those of sip110α (Figs. 3c, d, 4b, sip110α vs. sip85β + sip55γ).

Therefore, these results altogether confirm functionally that p110α/p85β and p110α/p55γ are the largely predominant PI3-K isoform complexes in HIEC cells, in addition to indicating that both complexes contribute in the activation of Akt-1 and a consequent promotion/maintenance of survival in these cells. However, these results also indicate that the p110α/p55γ PI3-K isoform complex performs the more predominant roles in Akt-1 activation and HIEC cell survival, than the p110α/p85β one.

### Selective engagement of PI3-K isoform complexes by integrin β1/Fak/Src-mediated suppression of HIEC anoikis

**Fig. 5:**
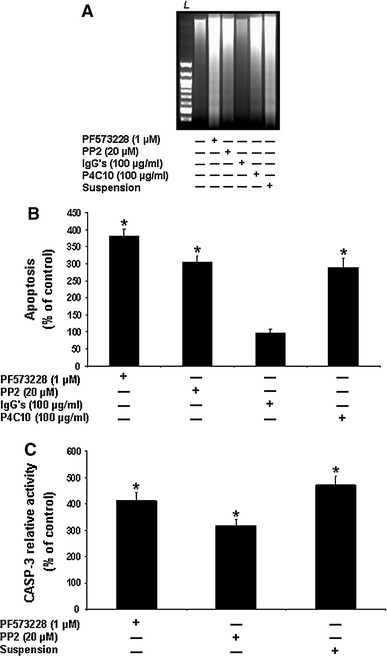
β1 integrin/Fak/Src-mediated signaling requirement for anoikis suppression in HIEC cells. **a** Representative (n ≥ 5) CAD-mediated DNA laddering assays from HIEC cell control cultures or cultures treated with PF573228, PP2, non-immune IgGs, the β1 integrin-blocking antibody P4C10, or kept in suspension in polyHEMA-coated dishes (suspension). *L* 100-bp DNA size markers. **b** HIEC cell cultures were maintained as in (**a**), except without the suspension treatment. ISEL was then performed. **c** HIEC cell cultures were maintained as in (**a**), except without the IgGs and P4C10 treatments. CASP-3 relative activity was then established, using the substrate Ac-DEVD-AMC, by comparison to controls. **b**, **c** Statistically significant (0.0001 ≤ *P* ≤ 0.001) differences between treated and control cultures are indicated by (*)

**Fig. 6:**
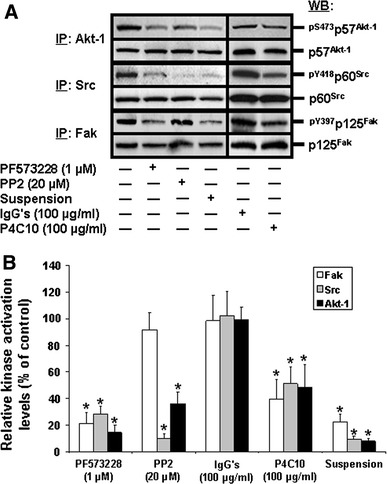
Integrin-mediated engagement of Fak, Src and PI3-K/Akt-1 in HIEC cell survival and suppression of anoikis. **a** Representative (n ≥ 3) WB analyses of Akt-1, Src and Fak IPs from HIEC cell control cultures or cultures treated with PF573228, PP2, non-immune IgGs, the β1 integrin-blocking antibody P4C10, or kept in suspension in polyHEMA-coated dishes (suspension). Specific antibodies for ^pS473^p57^Akt-1^, ^pY418^p60^Src^ and ^pY397^p125^Fak^, as well as for respective total protein forms, were used. **b** HIEC cells were maintained as in (**a**), except that the relative activation levels of Fak (*open columns*), Src (*grey columns*) and Akt-1 (*filled columns*) were established, then compared to controls. Statistically significant (0.0005 ≤ *P* ≤ 0.005) differences between treated and control cultures are indicated by (*)

As expected from our previous studies [28–33, 39], abundant CAD-mediated internucleosomal DNA fragmentation was observed in HIEC-6 cultures that were maintained in suspension (Fig. 5a, suspension), as well as in cultures exposed to the β1 integrin binding activity-blocking P4C10 antibody (Fig. 5a, P4C10), in contrast to control (adhering) cultures (Fig. 5a) and/or those exposed to non-immune IgGs (Fig. 5a, IgGs). Similarly, such DNA laddering was observed when the tyrosine kinase activities of Fak (Fig. 5a, PF573228) or Src (Fig. 5a, PP2) were inhibited. These observations were confirmed by ISEL (Fig. 5b) and CASP-3 activity (Fig. 5c) assays. The relative activation levels of Fak, Src and Akt-1 were then verified in both control and treated cultures. As previously reported [28–33, 39], the inhibition of Fak resulted in a significant down-activation of Fak itself (Fig. 6a, PF573228; 6b, PF573228, open column), of Src (Fig. 6a, PF573228; 6b, PF573228, grey column), and of Akt-1 (Fig. 6a, PF573228; 6b, PF573228, filled column), in a similar fashion as when cells were kept in suspension or exposed to the P4C10 antibody (Fig. 6a, b, suspension, P4C10). In the same vein, the inhibition of Src resulted in its own down-activation (Fig. 6a, PP2; 6b, PP2, grey column) and that of Akt-1 (Fig. 6a, PP2; 6b, PP2, filled column). However, such inhibition of Src had no significant impact on the activation levels of Fak (Fig. 6a, PP2; 6b, PP2, open column). Hence, while these results further support our previous demonstration that the PI3-K/Akt-1 pathway is engaged by integrin β1/Fak/Src signaling in the suppression of anoikis in HIEC cells [28–32, 39], these also suggest that the contributions of Src in such signaling may be primarily Fak-dependent—as observed in other cell contexts [6, 8, 14, 15].

**Fig. 7:**
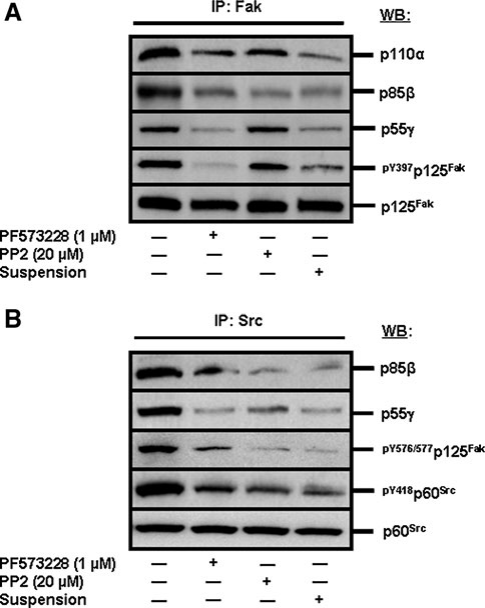
Selective engagement of PI3-K isoform complexes by integrin/Fak/Src-mediated signaling in HIEC cells. **a** Representative (n ≥ 3) WB analyses of the IP of Fak for the verification of association by co-IP of p110α, p85β, and p55γ, as well as verification of Fak activation, from HIEC cell control cultures or cultures treated with PF573228, PP2 or kept in suspension in polyHEMA-coated dishes (suspension). Specific antibodies for each PI3-K isoform probed, as well as for ^pY397^p125^Fak^ and p125^Fak^, were used. **b** Representative (n ≥ 3) WB analyses of the IP of Src for the verification of association by co-IP of p85β and p55γ, as well as verification of Fak-Src interactions and Src activation, from HIEC cell control cultures or cultures treated with PF573228, PP2 or kept in suspension in polyHEMA-coated dishes (suspension). Specific antibodies for each PI3-K isoform probed, as well as for ^pY576/577^p125^Fak^, ^pY418^p60^Src^ and p60^Src^, were used

Because both the p110α/p85β and p110α/p55γ PI3-K isoform complexes have been shown herein to contribute in the promotion of HIEC cell survival, albeit not equally (see previous section), we analyzed their engagement by integrin/Fak/Src signaling in the suppression of anoikis in HIEC-6 cells. To do so, we verified which PI3-K isoform complex is found in Fak/Src-mediated signaling cassettes by performing IP analyses (and verification of association via co-IP), following apoptosis/anoikis-inducing treatments and in comparison to untreated (control) cultures. We first set out to IP Fak—in this context, any presumptive signaling cassette partner associated with Fak would be co-IP, including Src and its own partners. Hence, in control cultures, a strong association of both p110α/p85β and p110α/p55γ complexes was revealed, as evidenced by the co-IP of p110α, p85β and p55γ (Fig. 7a). Accordingly, both the inhibition of Fak (Fig. 7a, PF573228) and induction of anoikis proper (Fig. 7a, suspension) resulted in the down-activation of Fak and a concomitant, extensive loss of association for all three PI3-K subunit isoforms. Interestingly, the inhibition of Src also caused a loss of association of p85β and p110α with Fak (Fig. 7a, PP2), yet failed to affect the association of p55γ (Fig. 7a, PP2), in addition to, once again, not impacting Fak activation (Fig. 7a, PP2).

Thus, in turn, we performed the same treatments but instead set out to IP Src. In this context, any presumptive signaling cassette partner associated with Src would be co-IP, including Fak and its own partners. To this effect, we again co-IP p85β and p55γ in control cultures (Fig. 7b). Accordingly, the inhibition of Fak and the induction of anoikis proper resulted in a down-activation of Src (Fig. 7b, PF573228, suspension), in a loss of Fak-Src interactions (as assessed by the Src-mediated phosphorylation of the Y576/577 residues of Fak [6, 14, 15, 32, 33, 35–37, 39]; Fig. 7b, PF573228, suspension), and a loss of association of p85β and p55γ (Fig. 7b, PF573228, suspension). Conversely, the inhibition of Src caused its own down-activation (Fig. 7b, PP2), a loss of interactions with Fak (Fig. 7b, PP2) and, consequently, a loss of association of not only p85β, but of p55γ as well (Fig. 7b, PP2).

Therefore, these results altogether indicate that both the p110α/p85β and p110α/p55γ PI3-K isoform complexes are engaged by integrin β1/Fak/Src signaling in the suppression of anoikis in HIEC cells. However, these results further reveal that such engagement of the two complexes in Fak/Src signaling cassettes is selective in nature. Indeed, the engagement of p110α/p85β is primarily Src-dependent (the engagement of which is itself primarily Fak-dependent), whereas the engagement of p110α/p55γ is primarily Fak-dependent (but Src-independent).

## Discussion

**Fig. 8:**
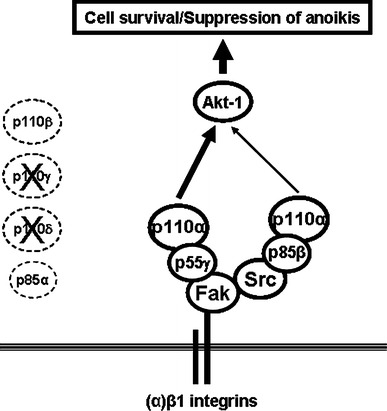
PI3-K isoforms and isoform complexes are selectively expressed, perform distinct roles in cell survival, and are selectively engaged by β1 integrin/Fak/Src-mediated signaling for the suppression of anoikis, in HIEC cells. Schematic drawing, which summarizes the results of the present study. p110α/p85β and p110α/p55γ are the largely predominant PI3-K isoform complexes in HIEC cells, whereas the individual isoforms p85α and p110β are expressed weakly, and p110γ and p110δ are not expressed. Only the p110α/p85β and p110α/p55γ complexes perform the critical functions of Akt-1 activation and subsequent maintenance of HIEC cell survival. However, the contributions of p110α/p55γ in Akt-1 activation and cell survival are significantly greater than those of p110α/p85β. Furthermore, p110α/p85β and p110α/p55γ are both engaged by β1 integrin/Fak/Src signaling in the suppression of anoikis; nevertheless, the engagement of p110α/p85β is primarily Src-dependent, whereas that of p110α/p55γ is primarily Fak-dependent (but Src-independent)

In the present study, we investigated the expression of PI3-K isoforms and their roles in the integrin β1/Fak/Src-mediated regulation of HIEC cell survival and suppression of anoikis. Herein, we demonstrate that p110α/p85β and p110α/p55γ are the largely predominant PI3-K isoform complexes in HIEC cells, whereas the individual isoforms p85α and p110β are expressed weakly, and p110γ and p110δ are not expressed. Concordantly, only the p110α/p85β and p110α/p55γ complexes perform the critical functions of Akt-1 activation and subsequent maintenance of HIEC cell survival. However, the contributions of p110α/p55γ in Akt-1 activation and cell survival are significantly greater than those of p110α/p85β. We also provide further evidence that the maintenance of HIEC cell survival and suppression of anoikis by β1 integrins is dependent on associated Fak signaling cassettes, in which Src is recruited. To this effect, we show that the p110α/p85β and p110α/p55γ PI3-K isoform complexes are selectively engaged by such integrin/Fak/Src signaling, whereby the engagement of p110α/p85β is primarily Src-dependent and that of p110α/p55γ is primarily Fak-dependent (but Src-independent). Hence, as summarized in Fig. 8, HIEC cells selectively express PI3-K R and C subunit isoforms, which translates into a selective expression of PI3-K R/C isoform complexes, which in turn results into isoform-distinct roles in the activation of Akt-1 and the promotion of HIEC cell survival and, additionally, in their selective engagement by β1 integrin/Fak/Src signaling in the suppression of anoikis.

It is now well established that PI3-K R and C isoforms can be distinctively expressed according to the cell type [16–23, 50]. In this respect, it is also accepted that one regulatory mechanism of the roles of PI3-K isoform complexes occurs at the gene expression level, in order to determine which isoform complexes are formed [16–23, 50]. This is well illustrated herein with regards to HIEC cells, as their selective expression profile of PI3-K R and C isoforms directly impacts on the constitution of the predominant class I PI3-K isoform complexes expressed by them (Fig. 8). Interestingly, our findings suggest that such a selective expression profile of PI3-K isoforms in HIEC cells is established not only via transcriptional regulation (e.g. weak mRNA levels, and consequently weak protein levels, for p85α and p110β), but furthermore via post-transcriptional and/or translational regulation (e.g. weak or strong mRNA levels for p110γ and p110δ respectively, yet absence of protein expression for both). Hence, these observations in HIEC cells emphasize the already acknowledged complex nature of the regulatory mechanisms that are responsible for the gene regulation of PI3-K R and C isoforms [16–23, 50], in addition to providing one more note added in proof to warrant further studies on the transcriptional and post-transcriptional/translational regulation of their expression. Such studies would be quite relevant to colorectal cancer (CRC), considering that although p110α is the predominant PI3-K C subunit in HIEC cells (this study), and that mutations of p110α conferring elevated/constitutive activity are found in one third of CRC tumors [34, 38, 40, 41], the expression of both p110α and p110β is nonetheless frequently elevated in CRC [34, 38, 40, 41, 51]. Similarly, p85α (but not, apparently, p85β) is likewise frequently elevated in CRC tumors [34, 38, 40, 41, 51]. More strikingly, a previous study reported that the predominant PI3-K isoform complexes in CRC cells are p110α/p85α and p110β/p85α [52], instead of p110α/p85β and p110α/p55γ as shown herein in HIEC cells. While the status of p55γ in CRC tumors and/or cells remains unknown, such findings in CRC altogether stand in stark contrast to our own in normal, non-transformed and non-immortalized HIEC cells—therefore underlying the need to investigate fully the expression, regulation and roles of PI3-K isoforms under the normal physiological context, in order to achieve a better comprehension of the aberrant expression and/or deregulation of these isoforms in cancer and cancer cell lines [16, 18–22, 38, 50, 51]. The same axiom would likewise apply with regards to other gastrointestinal disorders that display significant deregulation of IEC survival, such as inflammatory bowel diseases or necrotic enterocolitis [24, 25].

Although our knowledge of the regulation and roles of specific PI3-K isoform complexes remains poor [19–23, 50], there is nonetheless increasing evidence that such complexes can perform distinct functions in the regulation of various cell processes not only depending on the tissue context, but as well within the same given cell type [16–23, 50]. To this effect, the two predominant PI3-K complexes in HIEC cells (p110α/p85β and p110α/p55γ) not only contribute distinctively in the activation of Akt-1 and the maintenance of HIEC cell survival, but are furthermore engaged selectively by β1 integrin/Fak/Src signaling in the suppression of anoikis (Fig. 8). It is noteworthy that such functional identification of distinct PI3-K isoform complexes engaged by integrin/Fak/Src signaling, as well as the selective engagement by Fak and Src of said distinct isoform complexes, has never been observed or reported previously. Likewise, the identification of a p55γ-containing PI3-K complex engaged by β1 integrin/Fak-mediated signaling is novel. It is currently accepted that the R subunits are largely responsible for the specificity of engagement, as well as the distinctiveness of the roles enacted, of class I PI3-K isoform complexes [19–23, 50, 51]. As example, reports have shown that p85α, p85β and p55γ exhibit differential binding capacity to activated growth factor tyrosine kinase receptors (RTKs), such as those for insulin, epidermal growth factor (EGF) and platelet-derived growth factor (PDGF) [19–23, 50, 51, 53]. This is likely due first and foremost to individual structural and/or functional domain differences among R subunits, although this remains to be investigated more systematically [19–23, 50, 51]. For instance, while both p85β and p55γ bear two SH2 domains (one C-terminal—iSH2—for regulation of the C subunit, and one N-terminal—nSH2—through which recruitment/binding of the R subunit in a PI3-K complex occurs), p85β contains in its N-terminus one additional proline-rich motif, a BCR homology domain and an SH3 domain [19–22]. Therefore, such structural distinctions between p85β and p55γ may be largely responsible for their selective engagement observed herein by Src and Fak, respectively (Fig. 8). Furthermore, basic structural and functional differences between Fak and Src are also likely to contribute into such selective engagement of PI3-K isoform complexes. Depending on the cell context, Fak and Src have been shown able to directly recruit PI3-K [6–8, 13–15, 26, 36, 37, 54]. Alternately, Fak or Src can recruit PI3-K via various signaling cassette partners, such as the adaptors Shc or IRS-1 [6–8, 13–15, 26, 36, 37, 54]. Considering that Fak, or Src, or both, are often deregulated in cancer (including CRC) [6, 14, 36, 37, 39], further studies will be required in order to unravel the determinants that are responsible for the selective engagement of p110α/p85β and p110α/p55γ by Src and Fak, respectively, as reported herein. Additionally, roles in cell processes (other than survival and anoikis suppression) that p110α/p85β and p110α/p55γ may enact in HIEC cells remain to be investigated.

The relationship between selective PI3-K isoform expression and consequent distinct engagement/roles in cell survival shown in the present study is reminiscent of our previous findings in HIEC cells with regards to two other kinase isoform families—namely p38 and Akt. Indeed, HIEC cells express p38α, β and γ (but not p38δ), whereby the activation of p38β is antagonized by the PI3-K/Akt-1 pathway as it drives apoptosis/anoikis when activated, while the other two p38 isoforms play no role in either HIEC cell survival or death [29, 31, 32]. Similarly, HIEC cells express Akt-1 and -2 (but not Akt-3), whereby Akt-1 is β1 integrin/Fak/PI3-K-dependent for its activation and is required for cell survival, while Akt-2 activation is β1 integrin/Fak-dependent (but PI3-K-independent) and yet plays no role in HIEC cell survival or death [30–32]. To this effect, the siRNAs used herein and directed against either p110α, p85β or p55γ failed to affect Akt-2 activation (phosphorylation on the S474 residue) in any significant manner (data not shown). Hence, these previous observations concerning p38 and Akt isoforms, coupled to the current ones with regards to PI3-K isoforms and isoform complexes, further underlie the undeniable fact that the regulation of cell survival and anoikis constitutes a highly complex issue that implicates distinct mechanisms according to the cell type—at the very least [3–6, 9, 10]. However, the question now arises as to why p110α/p55γ performs the greater contributions, than p110α/p85β, to Akt-1 activation and HIEC cell survival, as well as why neither PI3-K isoform complexes influence Akt-2 activation. On the one hand, the precise determinants of the activation of each known Akt isoform specifically remain poorly understood [6, 16–18, 55, 56]. For instance, “Akt” activation can require its binding of PIP_3_ and the serine-threonine kinase activity of another PI3-K effector, PDK1, or can be altogether PI3-K-independent [16–18, 30, 55–57]. Although our findings herein confirm the requirement of PI3-K activity (specifically, that of p110α) for the activation of Akt-1 (but not Akt-2) in HIEC cells, we can now set aside another putative determinant of Akt-1 activation, namely the requirement for ILK (another PI3-K effector). Indeed, the siRNA-mediated expression silencing of ILK does not affect HIEC cell survival [45], as opposed to the suppression of Akt-1’s own activity through the forced expression of a dominant negative, kinase-dead Akt-1 mutant [30–32]. On the other hand, we have previously shown that the engagement of the PI3-K/Akt-1 pathway, including by β1 integrin/Fak/Src-signaling in the suppression of anoikis, is not only critical for HIEC cell survival, but furthermore translates into complex regulatory mechanisms of the expression and/or activity of cell survival determinants, such as individual anti- and pro-apoptotic Bcl-2 homologs [6, 28, 31, 32]. Therefore, additional studies will be required to elucidate the bases of Akt-1 activation by p110α/p85β and p110α/p55γ in HIEC cells, in addition to functionally identifying their roles in the regulation of Bcl-2 homologs, thus leading to a better understanding of their distinct contributions in HIEC cell survival.

## Conclusion

The present study allows for a clearer picture of the molecular determinants that are involved in the regulation of HIEC cell survival. Specifically, the findings herein provide evidence for the selective expression of PI3-K isoform complexes and a consequent distinct engagement of said expressed complexes by β1 integrin/Fak/Src-signaling, in turn translating into distinct contributions of these PI3-K complexes in the activation of Akt-1, the promotion of cell survival and the suppression of anoikis (Fig. 8). In addition to these novel findings, further studies should provide a greater understanding of the inherent complexities in the roles of PI3-K in the control of cell survival and apoptosis/anoikis not only within the normal physiological context of the epithelium of the gut, but as well within the physiopathological context of gastrointestinal disorders—such as CRC.
